# Health services research of integrative oncology in palliative care of patients with advanced pancreatic cancer

**DOI:** 10.1186/s12885-016-2594-5

**Published:** 2016-08-02

**Authors:** Jan Axtner, Megan Steele, Matthias Kröz, Günther Spahn, Harald Matthes, Friedemann Schad

**Affiliations:** 1 Forschungsinstitut Havelhöhe gGmbH, Kladower Damm 221, 14089 Berlin, Germany; 2 ihop Research Group, Queensland University of Technology, School of Public Health and Social Work, Victoria Park Road, 4059 Brisbane, Australia; 3 Krankenhaus Havelhöhe, Kladower Damm 221, 14089 Berlin, Germany; 4 Institut für Integrative Krebstherapie, Hans-Böckler-Str. 7, 55128 Mainz, Germany

**Keywords:** Pancreatic carcinoma, Palliative care, Integrative oncology, *Viscum album*, Mistletoe

## Abstract

**Background:**

Pancreatic cancer has a dire prognosis and is associated with a high mortality. Palliative patients have special needs and often seek help in integrative oncological concepts (IO) that combine conventional and complementary therapies. Nevertheless there are few recommendations regarding IO in current cancer guidelines. The aims of this study were to report on implementation of IO in everyday palliative care and to analyze patient survival in advanced pancreatic cancer.

**Methods:**

This multicenter observational study investigates the implementation of IO and length of survival of patients suffering from advanced pancreatic cancer (stage IV). We analyzed patient’s survival by employing multivariable proportional hazard models using different parametric distribution functions and compared patients receiving chemotherapy only, a combination of chemotherapy and *Viscum album* (VA) treatment, and VA treatment only.

**Results:**

Records of 240 patients were analyzed. Complementary therapy showed high acceptance (93 %). Most frequent therapy was VA treatment (74 %) that was often administered concomitantly to chemotherapy (64 %). Both therapies had positive effects on patient survival as they had significant negative effects on the hazard in our log-normal model. A second analysis showed that patients with combined chemotherapy and VA therapy performed significantly better than patients receiving only chemotherapy (12.1 to 7.3 month). Patients receiving only VA therapy showed longer survival than those receiving neither chemotherapy nor VA therapy (5.4 to 2.5 months). Our data demonstrates that IO can be implemented in the everyday care of patients without disregarding conventional treatment. Patients combining VA with chemotherapy showed longest survival.

**Conclusions:**

Our data demonstrate the importance and potential of health services research showing that IO treatment can be successfully implemented in the every-day care of patients suffering from advanced pancreatic cancer. Patients combining VA with chemotherapy showed longest survival. To address patients’ needs adequately, future cancer guidelines might increasingly include comments on complementary treatment options in addition to conventional therapies. Further studies should investigate the effect of complementary treatments on survival and quality of life in more detail.

## Highlights

We examined the implementation of integrative oncology in everyday care for palliative pancreatic cancer patients.

We used parametric models to analyze the hazard on patient survival.

Complementary therapies are chosen by a high number of patients.

Chemotherapy and complementary therapy with VA extracts were associated with lower hazards.

## Background

Pancreatic cancer is associated with a high mortality rate and thus remains a dire diagnosis for the patient. It is the eighth most frequent cancer diagnosis in Germany and is the fourth most common cause of death from cancer [1]. The nonspecific symptoms often lead to a delayed diagnosis of advanced cancer, resulting in a poor 5-year survival of six to nine percent [2]. Radical resection of the pancreas with adjuvant chemotherapy is the only curable approach [3], but only about 20 % of cases are resectable at diagnosis [4]. For palliative treatment chemotherapy has proven to be superior to best supportive care only [5]. Since the work of Burris et al. in 1997 [6], gemcitabine monotherapy has been the standard first line treatment [3, 7]. The combination of gemcitabine with the epidermal growth factor receptor inhibitor erlotinib; a chemotherapy of oxaliplatin, irinotecan, fluorouracil, and leucovorin (FOLFIRINOX) for patients with good performance status; and recently published albumin-bound paclitaxel followed by gemcitabine are alternative options that have shown promising results [8–10] and have found their way into current guidelines [3, 7, 11]. Nevertheless, with 6.24 months for gemcitabine/erlotinib and 11.1 months for FOLFIRINOX, the median survival remains poor. The shock of a cancer diagnosis and facing mortality usually results in a search for social support from family, friends or faith communities [12]. Patients have needs related to dealing with symptoms associated with disease or therapy, which strongly affect their well-being [12]. The major need that conventional medicine primarily focuses on is the need to cure, or at least to prolong survival [12]. Cancer guidelines mostly focus only on this outcome. Patients with advanced cancers often seek fulfillment of their other needs through complementary and alternative medicines (CAM) [13–15]. Patient’s expectations and motivations for using CAM are often not to cure, but to strengthen their immune system and to manage pain or other treatment-related side-effects [16]. The feeling of being active in their own treatment, with an associated level of self-control, is an important motivation for using CAM [12]. This seems to be of increased importance to patients with lengthy disease and treatment durations [17]. Additionally, sometimes patients see CAM as a last resort, providing hope in a situation of extreme distress [16, 17].

The Concerted Action for Complementary and Alternative Medicine Assessment in the Cancer field classifies such therapies as i) herbal products, ii) dietary approaches, iii) mind-body interventions, iv) manipulative and body-based practices or v) other CAM. The number of CAM-users has increased in recent years and is estimated to make up ~40 % of all cancer patients [18]. Simultaneous administration of CAM and conventional therapies without the knowledge of the physician always bears the risk of interference with the standard treatment [14]. In the concept of integrative oncology (IO) this is addressed and a comprehensive, patient centered treatment approach is planned and administered by a team of experts from different fields to avoid treatment interactions and to achieve the best possible outcomes [14]. IO attempts to combine the best of complementary and conventional therapies and to address patients’ needs beyond the alleviation of symptoms [14]. Although many patients evidently use CAM, it is almost not present in German guidelines [19] and is not documented in German official cancer registries [20]. In the following article we present data on patients with advanced pancreatic cancer documented by the Network Oncology, a conjoint clinical register of hospitals, practitioners and out-patient centers focusing on an IO concept [20]. We report on the amount and types of received complementary and conventional therapies and analyze patient’s survival.

## Methods

The Network Oncology (NO) has received a positive vote from the ethical committee of the Medical Association Berlin and has been described recently [20]. Using the NO clinical database, all consenting patients treated between August 2005 and November 2014 were thoroughly assessed and analyzed. Records of 459 patients with pancreatic carcinoma (ICD10 C25.*) were reviewed [20]. TNM stages or documented metastases were translated into UICC stages. Only palliative patients of stage IV at initial diagnosis (earliest recorded stage within a month of diagnosis) were included. Surgical interventions were coded according to the German procedure classification 2013 (OPS), the German modification of the international classification of procedures in medicine (ICPM) by the World Health Organization (WHO). Systemic therapies such as chemotherapy, targeted therapies or application of extracts of European mistletoe (*Viscum album*, VA) in the context of an integrative oncological setting were documented with start and end dates. According to the summary of medicinal product characteristics (SmPCs) VA is indicated in cancer therapy. The rationale is to improve health related quality of life (HRQL) by reducing therapy related symptoms and improving cancer related symptoms [21] with positive effects on coping, fatigue, sleep, exhaustion, nausea, vomiting and anxiety [22]. Non-pharmaceutical interventions (NPI) such as art therapies, nursing interventions or psychological therapies were documented as dummy variables. Some therapy types were grouped like nursing interventions (embrocation, compress and oil dispersion bath), movement therapies (physiotherapy, eurythmy therapy, ergo therapy), creative therapies (art therapy, music therapy, modeling and crafts therapy) and massage or lymph drainage. Censored patient survival was calculated from diagnosis date until recorded death or until last documented contact. Patients with no death date and no last contact date were excluded from the survival analyses. Kaplan Meier survival was calculated for all patients. To analyze how different factors influence the hazard on patient’s survival we employed proportional hazard models using different parametric distribution functions (log-normal, log-logistic, Weibull, Gompertz and extreme values). Tested parametric hazard models showed evident problems in dealing with long-term survivors. Therefore we excluded extreme values and censored our data to a maximum of 2 years of survival (730.5 days). We included the factors age, gender, chemotherapy and VA as variables as well as the individual number of NPIs and chose the model with the highest maximum likelihood.

To assess the effect of combined chemotherapy and VA therapy, we classified the data based on whether or not a patient received chemotherapy, and if they received VA therapy or not. VA therapy was defined as lasting more than 4 weeks, while the non-intensive group had VA therapy for less than 1 week or not at all. To avoid bias, patients that lived less than 4 weeks were excluded from these analyses. All analyses were conducted using R 3.1.2 [R Foundation for Statistical Computing, Vienna, Austria. http://www.R-project.org/]. Time event analyses used the R-packages ‘eha: Event History Analysis’ version 2.4-2 by Göran Broström and ‘survival: a Package for Survival Analysis in S’ version 2.37-7 by Terry M. Therneau.

## Results

Two hundred and forty (53.7 %) patients showed advanced cancer of UICC stage IV at first diagnosis. Median age was 68 (range 68–89 years). 124 patients were female (51.7 %). There was no significant difference in age between male and female patients. For nine patients a second, independent carcinoma was documented in the patient records. In seven cases this diagnosis occurred more than 1 year before the diagnosis of pancreatic cancer. Four of these cases were breast cancer diagnoses (ICD10 C50.*), the other localities that occurred only once were lung (ICD10 C34.*), prostate gland (ICD10 C61.*), and uterus (ICD10 C55.*). In two cases (ICD10 C34.* and C50.*) secondary diagnosis occurred 1 month and 4 months after the pancreatic diagnosis, respectively.

**Fig. 1:**
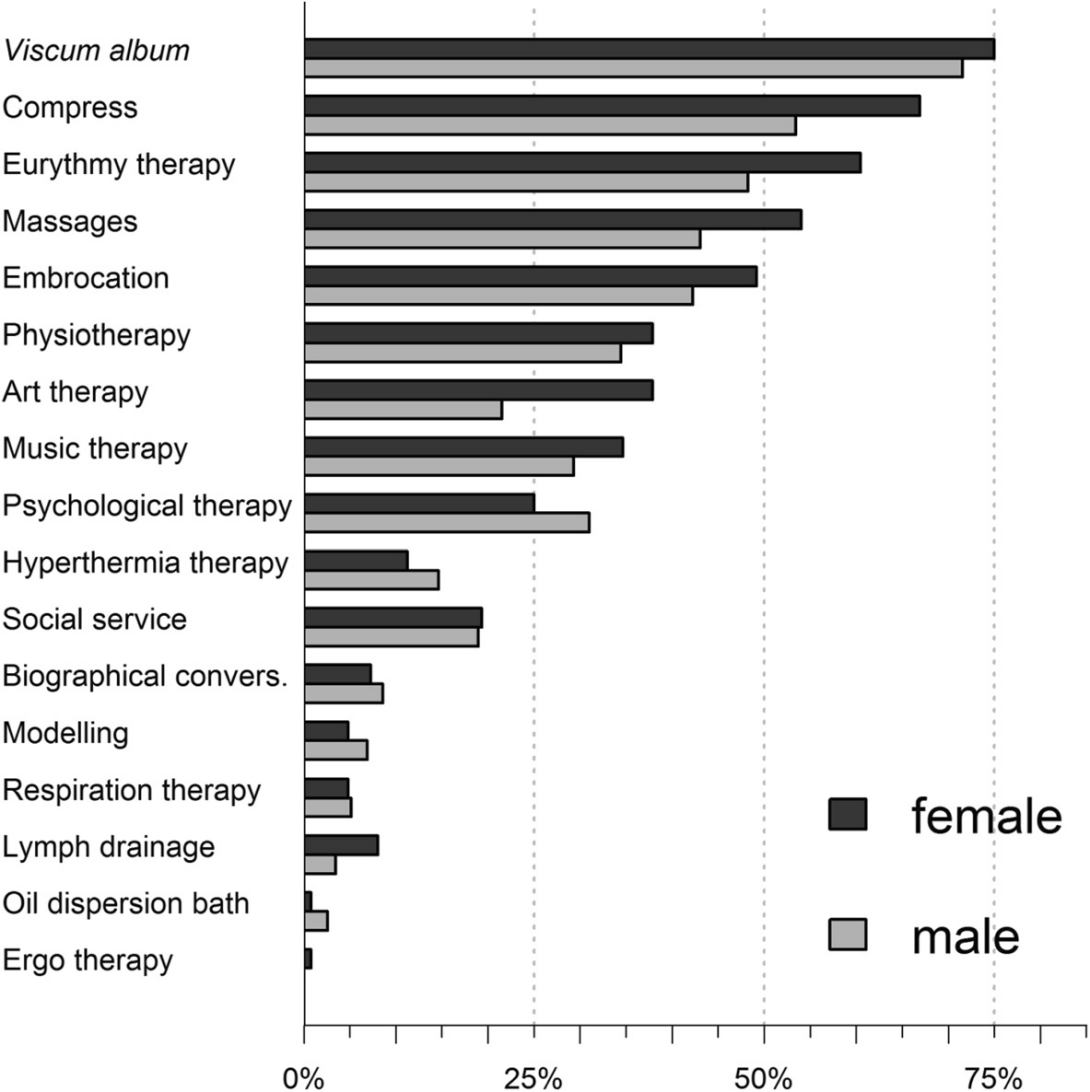
Relative frequencies of additional therapies in men and women. Dark grey bars denote female, light grey bars denote male patients

**Fig. 2:**
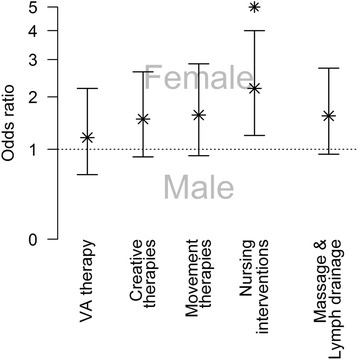
Odds ratios for male and female patients receiving a certain type of therapy. Asterisks denote statistical significance (*p* < 0.05)

Two hundred twenty-one patients (92.1 %) received a complementary therapy within an IO setting. 177 (73.8 %) received VA and 202 (84.1 %) received at least one NPI. Relative frequencies of patients receiving additional therapies are shown in Fig. 1. Amongst patients who received a NPI, the median number of different NPI therapies was five and did not differ between genders (W = 5640.5, *p* = 0.1676). 71.7 % of the patients had three or more NPI. Some therapy forms like nursing interventions were preferred by women compared to men (Fisher exact test p_Nursing_ = 0.006; Fig. 2). 89.2 % of the patients treated with VA received preparations subcutaneously, 35.2 % intravenously and 19.3 % by intratumoral application (Table 1).

**Table 1:** Number of patients receiving VA therapy

	Total		Female		Male	
VA therapy in general	176	73.3 %	93	75.0 %	83	71.6 %
Subcutaneous total	157	89.2 %	82	88.2 %	75	90.4 %
Intravenous total	62	35.2 %	32	34.4 %	30	36.1 %
Intratumoral total	34	19.3 %	13	14.0 %	21	24.4 %
Other total	4	2.2 %	3	3.2 %	1	1.2 %
Subcutaneous only	93	52.8 %	55	59.1 %	38	45.8 %
Intravenous only	6	3.4 %	5	5.4 %	1	1.2 %
Intratumoral only	6	3.4 %	2	2.2 %	4	4.8 %
Subcutaneous/intravenous	38	21.6 %	17	18.3 %	21	25.3 %
Subcutaneous/intratumoral	10	5.7 %	1	1.1 %	9	10.8 %
Intravenous/intratumoral	1	0.6 %			1	1.2 %
Subcutaneous/intravenous/intratumoral	15	8.5 %	8	8.6 %	7	8.4 %

The percentages in the first row refer to the total number of patients, other percentages refer to the number of patients receiving VA therapy

One hundred fifty-four patients (64.1 %) received chemotherapy with cytostatic drugs. 148 (96.1 %) of these patients received gemcitabine either as monotherapy or in combination with other drugs at some point. Thirty-two patients received a targeted therapy, mostly erlotinib in combination with gemcitabine (21 patients), or gemcitabine and oxaliplatin (2 patients), gemcitabine and tamoxifen (1 patient) or a monotherapy (11 patients) at some point during therapy. The monoclonal antibodies catumaxomab as monotherapy and bevacizumab in combination with gemcitabine were also used (each one patient). Five patients received radiation therapy. In two cases the radiated locality was the area of the pancreas, in two cases these were metastases along the thoracic vertebra and in one case it was the lumbar vertebra. 124 patients (51.6 %) underwent a surgical or endoscopic intervention (Table 2).

**Table 2:** Surgical and endoscopic interventions

	Interventions	Patients
Pancreatic interventions total	48	40
Inner drainage of the pancreas	1	1
Partial resection of the pancreas	19	19
Endoscopic intervention at the pancreatic duct	15	10
Anastomosis of the pancreatic duct	1	1
Other	12	12
Stomach interventions total	27	24
Intestine interventions total	29	23
Resections of small or large intestine	8	8
By-pass anastomosis of the intestine	5	5
Liver interventions total	15	15
Resections of the liver	14	14
Gall bladder or bile duct interventions total	105	49
Endoscopic interventions at the biliary tree	64	21
Other abdominal interventions total	43	35
Explorative Laparotomy	25	24
Other surgical or endoscopic interventions total	67	53
Port implantation	39	38
Splenectomy	4	4

**Fig. 3:**
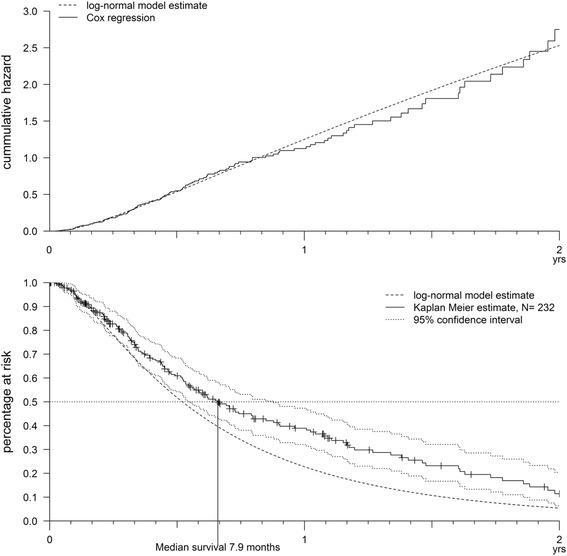
The hazard and the survival predicted by the log-normal model in comparison to the Cox regression and the Kaplan-Meier curve

Two hundred thirty-two patients were included in the right censored survival analyses and for 58.2 % of these we had exact death dates. The median survival was 7.9 months (CI_95%_ 6.6–10.5). For seven patients a survival of more than 2 years after diagnosis was documented. Five of these cases were censored with unknown outcome. Maximum survival was 7.5 years; five patients lived for more than 2 years, one for more than five and two for more than 7 years. The proportional hazard model with the highest log-likelihood had a log-normal baseline distribution (-888.78), followed by log-logistic (-890.17), Weibull (-892.68), Gompertz (-898.49) and extreme value (-899.40). Figure 3 shows the model fit of the hazard in comparison to the Cox regression and of the according survival function in comparison to the Kaplan-Meier curve. The fit of the hazard was good. The models with the highest likelihood all had in common that they allowed the hazard to first increase and then decrease, while the Weibull function only allows a monotonous incline. Nevertheless the model overestimates the hazard slightly from 11 months on. Accordingly the survival function slightly underestimates the real data. Model results showed that being male and of older age were associated with an increasing hazard, while chemotherapy, as well as VA, were associated with a decreasing hazard (Table 3).

**Table 3:** Proportional hazard models on patient survival

Covariate	w.mean	Coefficient	Exp (Coef)	SE (Coef)	Wald p
Intercept		1.613		1.730	0.351
Age	64.4	0.021	1.021	0.009	0.024
Male	0.5	0.423	1.540	0.179	0.016
Chemotherapy	0.8	-0.985	0.374	0.196	<0.001
VA therapy	0.9	-0.921	0.398	0.204	<0.001
Number of NPI	4.4	-0.010	0.990	0.032	0.765

‘w.mean’ is the weighted (against exposure time) means of the covariate; weighted relative frequencies of levels of factors

**Fig. 4:**
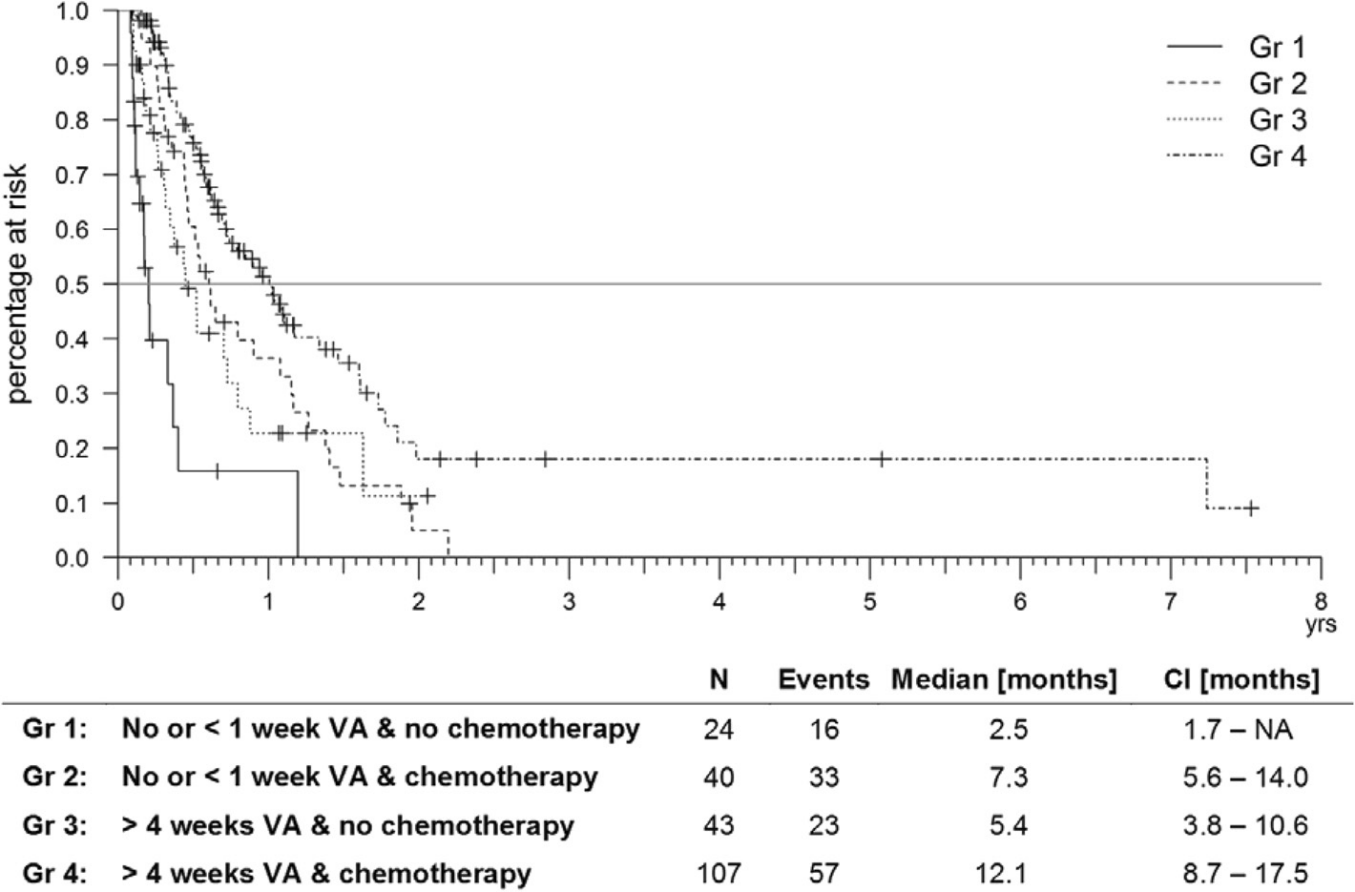
Kaplan Meier survival based on the UICC IV patients. Stratified for patients receiving no or less than 1 week of VA therapy and those who had it for more than 4 weeks, as well as for patients receiving chemotherapy or not

Analysis revealed a significant survival benefit for patients that received chemotherapy in combination with more than 4 weeks of VA (Gr 4) compared to patients having chemotherapy alone (Gr 2) (log rank test, *χ*^2^ = 6, *p* = 0.014, Fig. 4). Furthermore, the group receiving only VA (Gr 3) showed longer survival than the best supportive care group (Gr 1), that received no chemotherapy and no or almost no VA (log rank test, *χ*^2^ = 7.6, *p* = 0.006, Fig. 4). Table 4 summarizes and compares patient characteristics and NPI use by subgroup. Global tests showed significant differences in age, gender, total applied NPI and nursing interventions.

**Table 4:** Patients characteristics of the subgroups Gr1 – Gr4

	*N*	Gender		Age			Number of NPI			NPI				
		Male	Female	Min	Max	Median	Min	Max	Median	Total	Creative therapies	Movement therapies	Nursing therapies	Massages & lymph drainage
Gr 1	24	41 %	59 %	39	85	74	0	9	3	79 %	29 %	54 %	46 %	42 %
Gr 2	40	35 %	65 %	39	77	65	0	8	4	85 %	60 %	55 %	66 %	48 %
Gr 3	43	16 %	84 %	39	89	73	0	10	5	93 %	65 %	74 %	86 %	53 %
Gr 4	107	57 %	43 %	39	84	66	0	12	4	88 %	60 %	78 %	77 %	61 %
		*X* ^*2*^ test		Kruskal Wallis test			Kruskal Wallis test			*X* ^*2*^ test	*X* ^*2*^ test	*X* ^*2*^ test	*X* ^*2*^ test	*X* ^*2*^ test
		*X* ^*2*^ = 29.56		*X* ^*2*^ = 18.72			*X* ^*2*^ = 9.93			*X* ^*2*^ = 68.68	*X* ^*2*^ = 1.24	*X* ^*2*^ = 8.33	*X* ^*2*^ = 14.14	*X* ^*2*^ = 4.09
		***p*** **< 0.001**		***p*** **= 0.0003**			*p* = 0.0191			***p*** **< 0.0001**	*p* = 0.7426	*p* = 0.0396	***p*** **= 0.0027**	*p* = 0.2523

Gr1 patients received no chemotherapy and no or less than 1 week of VA therapy. Gr2 patients got chemotherapy but no or less than 1 week of VA therapy. Gr3 patients received no chemotherapy but a minimum of 4weeks VA therapy. Gr4 patients received a combination of chemotherapy and minimum 4 weeks of VA therapy. Non-parametric tests on group differences were made with a Bonferroni corrected α = 0.00625. Significant values *p* < 0.01 are shown in bold

## Discussion

The present health services research data demonstrate the use and acceptance of IO for palliative pancreatic cancer patients. Complementary therapies in IO are adjuncts to conventional cancer treatment, used primarily for symptom control, to enhance physical and emotional strength of patients during and after conventional care. Thus, IO aims to better address patients’ needs by combining the best of conventional medicine and CAM in a comprehensive way [14]. Claiming that only therapies used that were shown to be safe and effective, IO intends to prevent patients from harm by uncontrolled self-medication [14]. However, a common criticism is that many CAM methods are implemented in IO without sufficient risk-benefit analyses according to scientific standards [23]. Recent studies within the NO have addressed this issue and demonstrated safety profiles for key therapies used in IO settings [24, 25]. Patients included in this study tended to be younger compared to the German average of pancreatic cancer patients (men 70 year, women 76 years) [1]. This is in line with many studies reporting that younger age is often associated with CAM use [26]. However, demographic factors seem not to influence survival in pancreatic cancer patients in Germany [27]. A high percentage of our patients used CAM, especially NPI that intend to alleviate symptom burden and side effects, activate patient’s resources and improve patient’s quality of life by intensified care and attention. The latter might play a major role in our patient population, as three of the five most frequent NPIs were therapeutic nursing interventions. According to our analysis nursing interventions were significantly more preferred by women reflecting our previous results [20]. The importance of increased attention that focuses on patient’s needs has been demonstrated by Temel et al. [28], who showed significant improvements in HRQL, mood and survival in advanced lung cancer patients receiving an early palliative care in comparison to patients receiving standard care. Based on the individual needs of the patient, the early palliative care focused on an early psycho-oncological support and end of life care planning, including anticipation, prevention, and treatment of suffering. It was adapted from the National Consensus Project for Quality Palliative Care. Surprisingly, our data revealed that less than 30 % of patients chose psychosocial support (offered at the time of diagnosis) despite the vast burden associated with the diagnosis. This rather low rate may relate to heterogeneity in offer and acceptance of psychosocial support within the various participating centers and to incomplete documentation of complementary therapies in the early years of the study period.

Movement therapies were also common in our population and might contribute to patient improvement by giving the patient a feeling of still being able to lead an active life. Although positive emotional states are able to boost patients’ immune systems and have other benefits, it is likely that no amount of well-being can overcome such serious diseases like pancreatic cancer [29]. Other frequently used mind-body interventions were creative therapies that also address physical and emotional needs. Evidence suggests that such interventions might also have a positive impact on HRQL but more research is needed to draw a conclusion on their effectiveness [30, 31].

Generally, pancreatic cancer patients have a short survival time with an overall 5-year survival rate of between six to nine percent in Germany [1, 2]. Review of international literature and the SEER database, still indicates a 5-year survival of less than five percent [4, 32, 33]. In our data restricted to palliative patients, we documented a 2-year survival for 3.3 % of the patients. Our proportional hazard model demonstrated that older people and males faced a slightly higher risk of dying, whereas both chemotherapy and VA were associated with a relevant decrease of the hazard.

For patients diagnosed with already advanced cancer that received only best supportive care, a survival of 2.3 to 2.7 months is reported [33, 34], matching with our results of the stratified survival analysis. In our data this patient population lived significantly shorter compared to patients that received no chemotherapy but received VA for at least 4 weeks. Anticancer therapy with VA extracts is common in German speaking countries [35]. It has been reported to increase HRQL and attenuate adverse drug reactions to conventional therapies [36–38]. Whether VA can contribute to survival is the issue of a controversial debate [35]. A comprehensive overview on the mode of action of VA extracts is given by the following reviews [36, 39]. Results of our group on assessment of safety of intravenous and subcutaneous VA therapy in cancer patients revealed that it was safe with common dose-related expected effects and mild local (<5 cm) or temperature-related (<38 °C) adverse drug reactions and no serious adverse reactions [24, 25]. From our data we cannot conclude on the causality of VA and survival prolongation, but our result matches the result of Tröger et al. [40] in direction and magnitude. In their prospective randomized controlled study on VA treatment in patients with advanced pancreatic cancer they found a median survival of 4.8 months compared to 2.7 months in the best supportive care group. Our data also shows that VA alone seems not to be an equivalent to chemotherapy; but it might be a beneficial add-on to conventional chemotherapy as these patients showed longest survival. To evaluate the impact of VA as an add-on to standard chemotherapy on survival further confirmative randomized controlled trials are highly warranted. VA is well tolerated with gemcitabine [41] and recent studies have indicated a possible influence of VA on survival in patients with pancreatic carcinomas [42, 43]. This is in line with a systematic literature review on VA and cancer treatment [44]. A limitation of our study is that survival is likely to be underestimated. Whenever there was no exact death date documented we used the last documented date related to the patient as last contact date to calculate survival. The amount of censored data to the number of documented deaths is almost constant throughout the observed period. Nevertheless, missing death dates affect the calculation of survival. Additionally, as an observational study it suffers from potential missing or erroneous data and a heterogeneous patient population. Another limitation of this study it that the impact of the NPI in combination with conventional and/or VA treatment cannot be assessed within our data, since we are lacking systematic HRQL data. NPI are not well described in terms of prognostic relevance. However, there are positive effects of exercise on cancer-related fatigue [45] and overall survival in patients with breast-, and colon cancer [46, 47] and cognitive behavioral treatments and mindfulness orientated therapies in cancer-related fatigue. Whereas multimodal concepts are already established in chronic pain treatment [48, 49], their evaluation in cancer therapy is relatively new. In an exploratory analysis in breast cancer survivors with chronic cancer-related fatigue, psycho- and sleep education and eurythmy therapy where superior to aerobic training [50]. Patient-reported outcome measures (PROMS) have been developed for many years [51] and have been successfully applied in oncology [52–54]. While palliative chemotherapy in patients with end-stage cancer has been questioned to improve quality of life [55], in the future HRQL data and PROMS might contribute to the evaluation of the influence of single NPI-interventions and multimodal therapeutic approaches in cancer treatment.

Despite these limitations, our study provides an accurate picture of the current clinical use and the potential importance of IO in caring for pancreatic cancer patients.

## Conclusion

Our data demonstrates the importance and potential of health services research showing that IO treatment can be implemented in the every-day care of patients suffering from advanced pancreatic cancer. It also shows that IO treatment does not disregard conventional treatment. Our results suggest that IO therapies might have a beneficial effect on patient’s survival. However, there are few recommendations regarding IO in the current cancer guidelines [19]. As patients often use CAM, even without discussion with their practitioner [56], more attention should be given to this issue to help practitioners to address patients’ needs adequately and to prevent patients from harm by unqualified self-medication. These data demonstrate how health services research can reflect real life conditions and thus might contribute to ongoing discussion of treatment guidelines and direction of future research in this field.

## Abbreviations

CAM, complementary and alternative medicine; FOLFIRINOX, chemotherapy regimen with oxaliplatin, irinotecan, fluorouracil and leucovorin; HRQL, health related quality of life; ICPM, international classification of procedures in medicine; IO, integrative oncology; NPI, non-pharmacological intervention; PROMS, patient-reported outcome measures; SEER, Surveillance, Epidemiology, and End Results Program of the National Cancer Institute; TNM, Classification of Malignant Tumours of the Union for International Cancer Control; UICC, Union for International Cancer Control; VA, extracts of European mistletoe *Viscum album*; WHO, World Health Organization
